# How to verify the calibration of Goldmann tonometers

**Published:** 2012

**Authors:** Ismael Cordero

**Affiliations:** Clinical engineer Email: ismaelcordero@me.com

**Figure:**
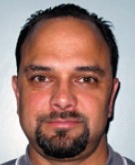
Ismael Cordero

A tonometer is an instrument for measuring the intraocular pressure (IOP), the fluid pressure inside the eye. It is an important test in the evaluation of patients with glaucoma, as damage to the optic nerve is more likely to occur in patients with high IOP. Most tonometers are calibrated to measure pressure in millimeters of mercury (mmHg).

Goldmann tonometry is considered to be the gold standard test for IOP and is the most widely accepted method. A disinfected prism is mounted on the tonometer head and placed against the cornea. The force applied to the tonometer head is then adjusted using a dial connected to a variable tension spring until the pressure in the eye can be determined from the force applied (see page 60 for a detailed description of how to use a tonometer).

Tonometers in busy clinics have been shown to lose accuracy within months of purchase or calibration by the manufacturer. They are more likely to deviate into the positive range, resulting in higher IOP measurements.

It is essential that all eye units develop protocols for calibration checks. Ideally, tonometers should be checked for calibration errors on a monthly basis by individuals who can be held responsible for ensuring their accuracy.

## Procedure

The following is the suggested user-level calibration verification procedure for a Goldmann tonometer. Calibration is done at dial positions 0, 2, and 6 (equivalent to 0, 20, and 60 mmHg, respectively).

Please note that this procedure is only intended to verify the accuracy of the instrument. If the tonometer is inaccurate at any of these dial positions, it should be returned to the manufacturer for recalibration.

## Before you start

- Insert the prism in the prism holder on the tonometer head and place the tonometer on the slit lamp.

## Calibration at dial position 0

**Figure 1:**
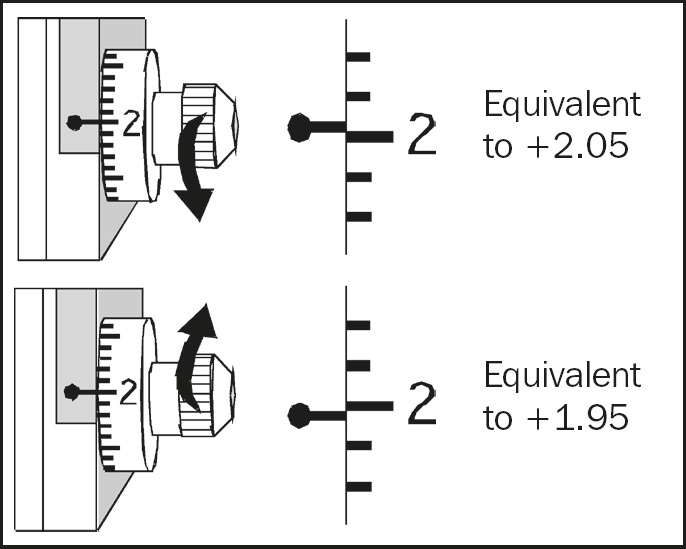
Calibration at dial position 0
- If the arm does not respond in the above way, the tonometer is inaccurate at dial position 0.

- At dial position 0, the feeler arm should be in free movement. If the dial is turned backwards a small distance (to the equivalent of position −0.05), the arm should fall towards the examiner. If the dial is turned forwards a small way (to the equivalent of position +0.05), the arm should fall towards the patient (Figure 1)

## Preparing to calibrate at dial positions 2 and 6

**Figure 2:**
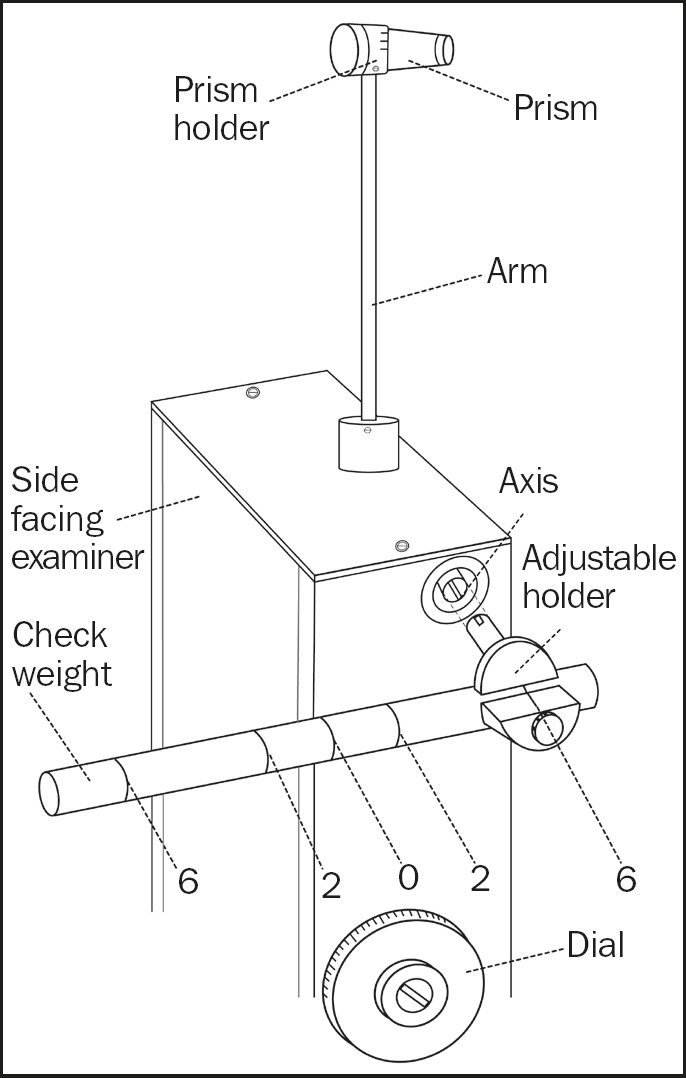
Construction of Goldmann tonometer

- To check dial positions 2 and 6, the check weight is used. The weight is in the shape of a bar and is normally found in the case with the tonometer prisms or in the drawer of the slit lamp. There are five line markings engraved on the weight. The middle marking represents 0, with 2 on either side of 0 and 6 towards the edges (Figure 2)
- Line up the adjustable holder with the lines representing either the ‘2’ or the ‘6’ mark on the weight. With the longer end of the bar facing the examiner, slide it into the axis on the side of the tonometer and push it all the way in (Figure 2).

## Calibration at dial position 2

**Figure 3:**
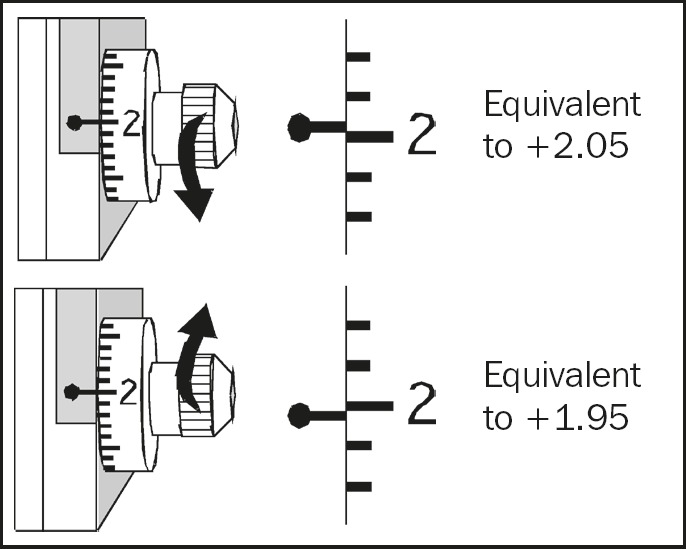
Calibration at dial position 2
- If the arm does not respond in the above way, the tonometer is inaccurate at dial position 2.

- Check that the adjustable holder is lined up with the line representing the 2 mark (Figure 2) and that the longer end of the bar is facing the examiner.
- Set the dial to position 2. As with dial position 0, the feeler arm should be in free movement. If the dial is turned backwards a small way (to the equivalent of position +1.95), the arm should fall towards the examiner. If the dial is turned forwards a small way (to the equivalent of position +2.05) the arm should fall towards the patient (Figure 3)

## Calibration at dial position 6

**Figure 4:**
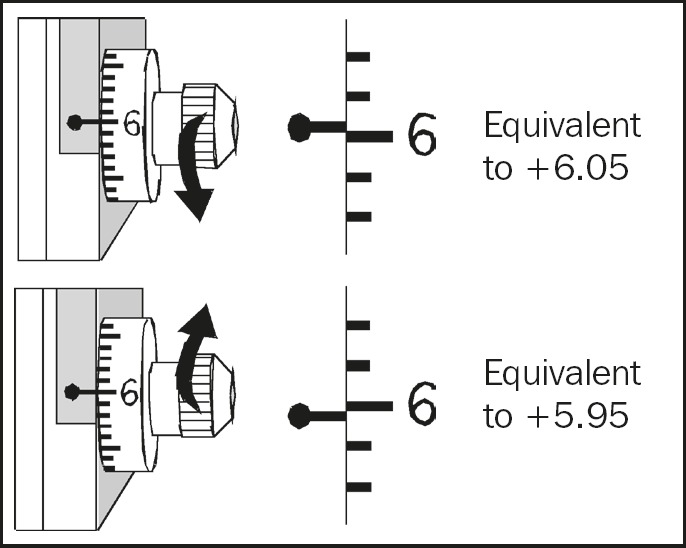
Calibration at dial position 6

- Check that the adjustable holder is lined up with the line representing the ‘6’ mark (Figure 2) and that the longer end of the bar is facing the examiner.
- Set the dial to position 6. If the dial is turned backwards a small way (to the equivalent of position +5.95), the arm should fall towards the examiner. If the dial is turned forwards a small way (to the equivalent of position +6.05) the arm should fall towards the patient (Figure 4)
- If the arm does not respond in the above way, the tonometer is inaccurate at dial position 6.

